# GAME (Goals - Activity - Motor Enrichment): protocol of a single blind randomised controlled trial of motor training, parent education and environmental enrichment for infants at high risk of cerebral palsy

**DOI:** 10.1186/s12883-014-0203-2

**Published:** 2014-10-07

**Authors:** Catherine Morgan, Iona Novak, Russell C Dale, Andrea Guzzetta, Nadia Badawi

**Affiliations:** School of Medicine, University Of Notre Dame Australia, PO Box 6427, Frenchs Forest, NSW 2086 Australia; Cerebral Palsy Alliance Research Institute, University of Notre Dame Australia, PO Box 6427, Frenchs Forest, NSW 2086 Australia; Department of Neurology, Children’s Hospital at Westmead, University of Sydney, Locked Bag 4001, Westmead, NSW 2145 Australia; Department of Developmental Neuroscience, Stella Maris Scientific Institute, Pisa, Tuscany Italy; Grace Centre for Newborn Care, Children’s Hospital at Westmead, University of Sydney, Locked Bag 4001, Westmead, NSW 2145 Australia

## Abstract

**Background:**

Cerebral palsy is the most common physical disability of childhood and early detection is possible using evidence based assessments. Systematic reviews indicate early intervention trials rarely demonstrate efficacy for improving motor outcomes but environmental enrichment interventions appear promising. This study is built on a previous pilot study and has been designed to assess the effectiveness of a goal - oriented motor training and enrichment intervention programme, “GAME”, on the motor outcomes of infants at very high risk of cerebral palsy (CP) compared with standard community based care.

**Methods/design:**

A two group, single blind randomised controlled trial (n = 30) will be conducted. Eligible infants are those diagnosed with CP or designated “at high risk of CP” on the basis of the General Movements Assessment and/or abnormal neuroimaging. A physiotherapist and occupational therapist will deliver home-based GAME intervention at least fortnightly until the infant’s first birthday. The intervention aims to optimize motor function and engage parents in developmental activities aimed at enriching the home learning environment. Primary endpoint measures will be taken 16 weeks after intervention commences with the secondary endpoint at 12 months and 24 months corrected age. The primary outcome measure will be the Peabody Developmental Motor Scale second edition. Secondary outcomes measures include the Gross Motor Function Measure, Bayley Scales of Infant and Toddler Development, Affordances in the Home Environment for Motor Development – Infant Scale, and the Canadian Occupational Performance Measure. Parent well-being will be monitored using the Depression Anxiety and Stress Scale.

**Discussion:**

This paper presents the background, design and intervention protocol of a randomised trial of a goal driven, motor learning approach with customised environmental interventions and parental education for young infants at high risk of cerebral palsy.

**Trial registration:**

This trial is registered on the Australian New Zealand Clinical Trial register: ACTRN12611000572965.

## Background

Cerebral palsy (CP) is the most common physical disability of childhood with a prevalence of 2.1/1000 live births [1]. Late diagnosis, conservative “wait and see” monitoring and late referral to early intervention is the prevailing norm for two main reasons. First, because only half of infants with CP have clearly identifiable risks in the newborn period, for example prematurity or neonatal encephalopathy (NE) [2], and second, because not all infants with prematurity or NE will go on to have CP. Wait and see monitoring can mean brain injured infants do not always receive intervention in the most crucial period of brain development [2]. Furthermore children with CP reach approximately 90% of their gross motor potential by age 5 (or younger for more severely impaired), but for 40% of this critical window the ultimate severity of their condition is largely unknown [3], however severity itself is a likely predictor of responsivity to early intervention. The field of neuroscience has repeatedly demonstrated the plasticity of the infant brain and persistence of neurogenesis and activity- dependent plasticity are two of the basic mechanisms at work [4]. Intervention for infants with brain injuries aims to optimise these neuroplastic mechanisms.

In recent years, research into the predictive validity of Prechtl’s Qualitative Assessment of General Movements (GMs) has allowed earlier diagnosis of high risk of CP to be reliably made at 3 months of age [5,6]. GMs is now the gold standard tool for early diagnosis of CP because of higher specificity and sensitivity than other traditional tests such as neurological examinations, cranial ultrasound and MRI [7]. For the preterm population, the combination of GMs and evidence of white matter injury on MRI predicts CP at 3 months with 100% accuracy [6].

### Early intervention and early enrichment

Early intervention (EI) studies have typically not used this combination of assessment tools to recruit homogenous samples of infants at high risk of CP. Rather heterogeneous infants are included in EI studies and labelled “high risk” because they were preterm, display delayed development or had complex social issues [8]. In many of these studies the proportion of children who actually go on to be diagnosed with CP are relatively small resulting in underpowered type II trials for CP. As a result it is virtually impossible to ascertain the effects of EI on the motor outcomes of infants with CP. Most systematic reviews conclude that EI approaches currently in use for CP do not have any effect on motor outcomes greater than what would be expected as a result of maturation [9,10]. It is important to note, however, that evidence for the effectiveness of general EI to improve cognition is well established for the more heterogeneous “high risk” groups [11].

It remains to be determined whether intervention approaches that are goal-oriented and involve active motor training [12,13] currently used in older children with CP are actually applicable to infants with a small emergent motor repertoire. In addition, what “active ingredients” from EI approaches are vital to maximise developmental outcomes?

Environmental enrichment (EE) has been proven to enhance neuroplasticity and promote memory and motor function in animal studies [14] but the effect in humans is less understood. In animal studies, an EE is defined as one that facilitates enhanced cognitive, motor and sensory stimulation. Although there is no agreed parameters for enrichment, these animal housing conditions typically include high levels of complexity and variability with arrangement of toys, platforms and tunnels being changed every few days to promote motor learning and memory. The motor opportunities afforded by EE are a critical success factor.

Translating these ideas into the human context is complex. Much more is known about the detrimental impact of deprivation (under-enrichment) on child development than is known about what constitutes enrichment for infants raised in “expected environments” [15]. Thus a continuum of enrichment is implied, but has not been well explained in terms of the type or amount of enrichment required for children who are not typically developing. One recent systematic review [8] has demonstrated a small positive effect on motor outcomes for infants at high risk of CP when the utilised interventions are based on principles of environmental enrichment. The enhanced plasticity mechanisms present in the infant brain allow it to be more strongly influenced by the environment than adult brains, so furthering our understanding of what constitutes enrichment for brain injured babies is important [16,17].

In children with CP the key environmental factors which influence motor development are yet to be determined, however clinical and neuroscience do provide a clear rationale for the urgent need for the development of EI programmes that focus on EE strategies to improve motor outcomes in these children [18]. Ulrich’s [19] recent review discusses the opportunities for the development of early intervention programs which link neuroscience with clinical science and states in her summary, “A growing body of basic and clinical science results suggest we are missing the boat on opportunities for infants with motor disabilities if we do not develop more empirically based protocols to use very early in life in order to optimize developmental outcomes” [19], p10.

We have developed such a protocol, “GAME”, based upon the principles of motor learning and widely accepted EI frameworks including family centred practice [20] and the ecological framework [21]. Data from our recent pilot randomised controlled trial RCT (n = 13) indicates that GAME, a goal-oriented, intensive motor training programme that actively involves parents and includes EE strategies, could be effective in advancing the motor trajectories of infants at high risk of CP [22]. After 12 weeks, GAME intervention infants (n = 6) had an 8.05 point advantage on the Total Motor Quotient of the Peabody Developmental Motor Scales – second edition compared to infants who received standard care therapy (n = 7). Although small, the pilot study confirmed feasibility of recruitment and randomisation procedures, and enabled confirmation of outcome measures and the sample size required for a larger RCT of GAME intervention. This proposed study will address this gap in the literature.

### Objective

The aim of this study is to evaluate whether a goal oriented, intensive motor training programme with EE strategies (GAME) is more effective than current standard care practices in influencing the early motor development of infants at high risk of CP.

## Methods

A single blind RCT with 2 parallel groups will be conducted to evaluate the efficacy of GAME compared to standard care. The outcomes of this trial are the infant’s motor function after 16 weeks of intervention and at 12 and 24 months corrected age, home enrichment, parent perception of and satisfaction with their child’s performance and parental well - being.

We hypothesise that:

1. Infants diagnosed with CP or at risk of CP that receive GAME intervention will have higher short term (after 16 weeks of intervention) Peabody Developmental Motor Scale (PDMS II) scores than infants that receive standard care
2. Infants diagnosed with CP or at risk of CP that receive GAME intervention will have higher long term (at 1 year of age) scores on the PDMS II scores than infants that receive standard care.
3. Infants diagnosed with CP or at risk of CP that receive GAME intervention will have higher Gross Motor Function Measure (GMFM) scores than infants that receive standard care at 1 year of age.
4. Infants who have received GAME intervention will have sustained higher PDMS –II scores long term (at 24 months) compared with infants who have received standard care.

### Study sample and recruitment

Thirty infants will be recruited from their treating institution, community physician or local therapist. The infants will be recruited in and around Sydney, NSW Australia. Seven NICUs and the Cerebral Palsy Alliance will actively recruiting to this study although infants may be referred from any source. Study sites are listed in the Appendix.

All parents of eligible infants will be informed about the study only after they have had discussions with their medical team regarding the high risk status of their child, or a confirmed diagnosis of CP. Families will be given a site specific information sheet regarding the purpose and design of the study and have opportunity to speak with investigators before consenting to the study. Parents who do not wish to consent to the study will be offered standard community based therapy.

After consent is obtained, prior to randomisation, the investigators will visit the family at home to complete all baseline assessments and collect demographic and perinatal data. MRI and medical data will be obtained from the infant’s medical record.

**Figure 1:**
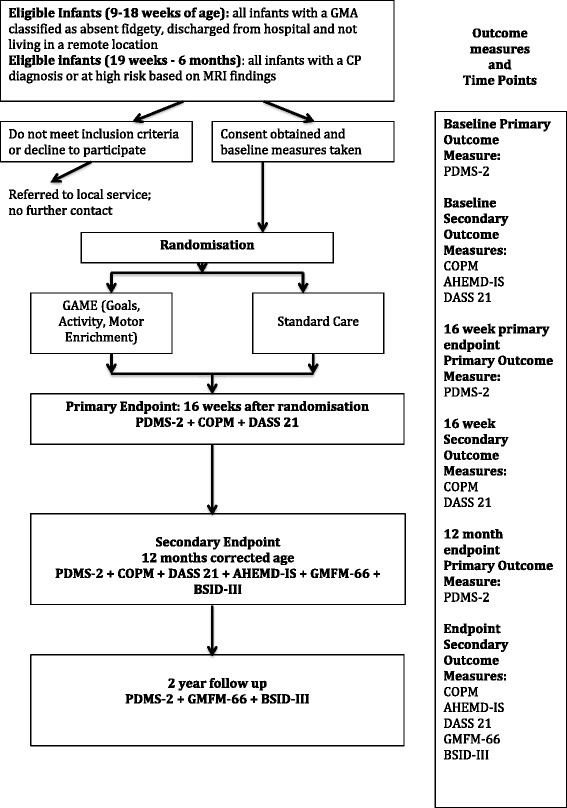
**Consort flow chart.**

The Human Research and Ethics Committees of the Sydney Children’s Hospital Network (SCHN), Cerebral Palsy Alliance (CPA) and the University of Notre Dame Australia (UNDA) have approved this study. The experimental design including time points and outcome measures are depicted in the CONSORT [23] flowchart (Figure 1).

### Inclusion criteria

Infants aged between 3 and 6 months (corrected age) with a diagnosis of CP or at high risk of CP are eligible for the study. Infants referred between 9–18 weeks post term age (PTA) will be screened using the General Movements Assessment (GMs). At least 2 certified assessors blinded to the infant’s history will score the GMs videos. Infants with abnormal general movements (absent fidgety) are eligible for enrolment, ie 95% high risk of CP. Where assessors disagree, a third blinded assessor will be required to assess the video.

Infants over 18 weeks corrected age up to 6 months of age, outside the window of reliable GMs assessment, will be included on the basis of a confirmed CP diagnosis and/or abnormal neuroimaging as described by Krageloh-Mann [24].

Imaging commonly associated with CP include:

1. Periventricular Leucomalacia (PVL) and cystic PVL
2. Intracranial Haemorrhage
3. Periventricular infarction
4. Lesions of the basal ganglia and thalamus
5. Unilateral parenchymal injury eg middle cerebral artery infarction
6. Cortical malformation

A pediatric neurologist blinded to group allocation will confirm MRI features.

### Exclusion criteria

Infants otherwise eligible but with severe genetic abnormalities, or not discharged from hospital, or residing in remote areas not accessible to the research team will not be eligible for the study.

### Sample size

The planned study sample size (n = 30; 15 per group) has been estimated from a power calculation based on our pilot data using motor composite scores of the PDMS-2, with an alpha value of 5% and power of 80%, using a minimal clinically important difference of 10%, accounting for a 20% dropout rate.

### Randomization process

After informed consent and baseline measures are taken, an officer not connected with the study will randomise participants at a separate location using a pre-prepared random assignment schedule stored within 30 concealed opaque envelopes generated using computer generated random numbers. The Primary Investigator will be informed by the independent randomisation officer of group allocation and will inform parents. Twins will be randomised together due to the nature of the intervention.

#### Blinding arrangements

The independent assessors will be blinded to group allocation and will carry out all assessments after randomisation. Assessments of the child’s movement for the primary outcome measure and GMFM-66, will be completed via scoring from video. Other secondary outcome measure assessments will be conducted over the phone, via home visit or parent self report, as per the test and clinical conventions. Research Assistants from the Cerebral Palsy Alliance Research Institute and trained physiotherapists and/or occupational therapists will score the measures as the blind assessors.

It is not possible for either the participating families or those conducting the intervention to be blinded in this trial due to the nature of the intervention.

### Intervention

#### Therapists

Investigators CM, an experienced physiotherapist and IN, an experienced occupational therapist are the primary therapists providing the GAME intervention to maximise fidelity of the intervention. If a speech pathologist or family support worker is required based on identified family goals this will be provided. Infants in the standard care group will receive services from local therapists according to the centre’s protocol. Typically in Sydney this would include physiotherapists and/or occupational therapists. Some sites offer a multidisciplinary team approach while others a keyworker model with a primary therapist.

### Interventions

**GAME** is a therapy intervention based on contemporary motor theory. This intervention approach has been previously described in a small pilot RCT that tested the feasibility of GAME [22]. GAME intervention consists of three components: goal-oriented intensive motor training, parent education, and strategies to enrich the child’s motor learning environment. Although described as distinct aspects of GAME, these components are fully integrated into therapy sessions with the emphasis on any particular component varying from session to session.

#### Game part 1

##### Goal-oriented intensive motor training

Families collaborate with the therapists to determine a set of goals for their child’s development [25]. Typically the goals would relate to motor development but might also include health related concerns known to affect development such as sleeping and feeding. The therapist plays an important role in helping parents set realistic and appropriately time framed goals. As goals are attained the family and therapist work together to develop new goals. These parent identified goal areas are targeted for practice during therapy sessions and built into a home programme (HP).

The motor learning component of the intervention is based on the principles of motor learning and dynamic systems theory [26,27]. Therapist assessment of the relative contributions of weakness, selective motor control and altered tone to difficulties in goal achievement are discussed with the family and solutions are identified and tried [28]. Parents are encouraged to use their knowledge of their child’s play preferences to elicit self-generated motor activity. Minimal manual guidance is provided when required and withdrawn as soon as the child has the idea of the movement or begins to demonstrate the ability to recruit a successful muscle action or sequence. Parents are coached in understanding “missing components” of the desired action and problem solve with the therapists ways of simplifying the task to enable at least part task attainment.

Motor tasks are scaffolded, so that the infant can always actively complete at least a part of the task [29]. As performance improves, the motor challenge is increased by altering the task or environment to encourage problem solving. Manual assistance is reduced or withdrawn as soon as the infant demonstrates self-initiated progress with the task; ensuring self-generated motor activity is promoted in all practice sessions. Once a motor skill is learned, variability of practice is introduced to increase the complexity and generalizability of the skill [30]. Early weightbearing and sit to stand from the parents’ lap are routinely included for each infant even if standing is not identified as a specific goal. Rehabilitation research in older children and adults with brain injuries suggest that functional weight bearing exercises can both improve motor control and provide strength training [26]. Given that the expected impairments of CP include weakness and reduced selective motor control, early activation of muscles of the lower limb using both concentric and eccentric exercise could enhance the development of upright mobility. Similarly, practice of reaching and grasping a variety of objects is also a standard part of motor training for all infants in order to expose the infants who are expected to be delayed, to a variety of objects to advance grasp and reach behaviours [31]. Modified constraint induced movement therapy and/or bimanual training is used when asymmetrical hand function is evident.

Practice schedules are discussed and designed based on family time constraints. A written HP, illustrated with photographs and related to parent identified goals, weightbearing and reach and grasp is provided. The HP describes parenting strategies, environmental enrichments and child-activities as per published guidelines on effective home programmes [32]. Activities in the HP are organised into those in which the carer plays an active role and those where practice can be “set up” for the infant to carry-out independently. The HP is updated as goals are attained.

#### Game part 2

##### Parent education

Parent education is known to be an important component of early intervention that is grounded in family centred practice [33]. Since most of the infant’s active practice opportunities are provided in the child’ daily routines, parent education is vital [34]. In GAME intervention, parents are coached to identify their child’s voluntary attempts to move and self-regulate, plus understand the usual trajectory of emergent motor skills and how to stimulate progress. Parents are trained in simple motor task analysis and coached in appropriate strategies to enhance their child’s development both at a specific goal level and in general early learning and development principles. Parents are taught to optimise the best use of their infants’ “awake” time and the naturally occurring opportunities for learning. Learning optimisation includes both parent-directed and structured practice of desired motor tasks, where the parent role is integral to the child’s learning (e.g. creating repetitions) and constructing opportunities for independent play (e.g. playing alone with motor enriching toys set up for the child). Parents are encouraged to both observe the therapist eliciting a motor behaviour from the baby and to attempt it themselves. Specific feedback, in a warm and supportive context, is given to parents to enable them to tease out why some attempts were successful for the baby and others weren’t. As new motor skills emerge parents are coached in strategies to increase the challenge of the task; for example removal of support or the introduction of more complex toys. The importance of allowing trial and error during practice is discussed and parents are encouraged to devise their own activities to enhance goal attainment. Prognostic information is given when possible as well as evidence based information regarding sleeping, feeding and responsive parenting.

#### Game part 3

##### Environmental enrichment

It is clear that many aspects of a child’s environment influence his or her motor, cognitive and social-emotional outcomes. Parental responsivity, a variety of daily experiences, equipment use and the structure of the physical space are all known to influence child development [35-37]. In GAME, all visits are conducted within the family’s home and deliberate attention is paid to aspects of the home environment to enhance developmental outcomes. This enrichment includes assistance in setting up motor enriched play environments to promote child self-generated movements, exploration and task success. This includes instruction in careful toy selection “matched” to the desired motor task, plus physical set up of areas for practicing and repeating activities related to the identified goal areas, weightbearing, and reaching and grasping tasks. Conventional baby equipment (e.g. highchairs, toys) already purchased by the family is used wherever possible. The whole environment for motor learning is taken into account and therefore intervention may also include: (a) evidence-based early learning stimulation and role modelling to enhance cognitive and language development (e.g. reading books to children, limiting passive television watching); (b) optimising sleep hygiene; and (c) feeding interventions (e.g. anti-reflux medications) to ensure adequate caloric nutrition and pain-free backdrops for learning. The importance of variable daily experiences for infants is deliberately addressed and support given when parents articulate difficulty leaving the house. Siblings and extended family members are also actively encouraged to take part in the HP and therapy sessions to promote: family knowledge; family acceptance; family wellbeing; repetition of learning opportunities; and provide a natural source of varied social interaction for the infant. Parent well-being is openly discussed and support given to parents to access appropriate services when required.

Home visits from the GAME treating therapists are offered weekly initially and then frequency of intervention negotiated with each family around their preferences, availability and family resources required to carry out the intervention with fidelity. Visits are approximately 60 to 90 minutes duration.

### Standard care

“Standard care” (SC) describes the current follow-up and/or therapeutic interventions used when an infant deemed at high risk of CP is discharged from hospital in New South Wales Australia. It is not possible to standardise the frequency, intensity or type of interventions received in the SC group. Approaches used are varied and might include neurodevelopmental therapy, the developmental skills approach, group therapy or motor learning approaches reflective of the current EI literature base. Most therapists include parent education on positioning and handling and suggested home activities within the therapy programme. In the pilot study, SC therapy was offered approximately monthly but ranged from fortnightly to 3 monthly. In order to monitor the mode, frequency and intensity of intervention received by those in the standard care group as compared to the GAME group, all parents will be asked to keep a “log book” so that these relevant parameters can be compared between the groups. Similarly since the actual interventions provided in SC are likely to vary between services, history taking will include information gathering regarding the type of interventions used.

### Outcome measures and procedures

#### Peabody Developmental Motor Scales -Second edition (PDMS-2)

The PDMS-2 [38] is the primary outcome measure in this trial and is a frequently used assessment of motor skills. This test is standardised and normed for children aged from birth to 6 years and has been validated for use as a discriminative measure. Two studies have demonstrated that it is responsive to change in the CP population for both infants [39] and toddlers [40]. It has demonstrated concurrent validity with the GMFM [41] and the Bayley [42]. PDMS-2 assessments will be obtained at baseline, 16 weeks after therapy has commenced and at 12 months and 2 years. Assessments will be blind scored from video.

#### Gross Motor Function Measure (GMFM)

The GMFM [43] is a criterion-referenced tool that is widely accepted as the gold standard for gross motor assessment in children with CP. There are a total of 5 dimensions measured including rolling, sitting, creeping, standing and walking. Infants will be videoed during the assessment and blind raters will score from the video using the appropriate manual. The GMFM- 66 will be used in this study at the secondary endpoint, (12 months) and at the 2-year follow up.

#### Canadian Occupational Performance Measure (COPM)

The COPM [44] is an individualised criterion referenced measure of performance of a self-selected range of activities. Functional problem areas are identified, prioritised and rated for performance and satisfaction via a semi-structured interview. The COPM will be used to prioritise goals and measure change in performance and satisfaction. The COPM will be used at baseline, 16 weeks after therapy has commenced and at 12 months. Data will be collected via face to face or phone interview by independent raters.

#### Affordances in the Home Environment for Motor Development-Infant Scale (AHEMD-IS)

The AHEMD-IS [45] is a measure of the quality and quantity of motor enrichment opportunities available to a child within the home environment. This tool has demonstrated validity and reliability in the toddler format. Data is collected via a parent self report on a standardised questionnaire. A total raw score is calculated. This measure will be taken at baseline and at 12 months.

#### Depression, Anxiety and Stress Scale-21 (DASS)

The DASS-21 [46] is an adult self-report designed to measure the emotional states of depression, anxiety and stress. It is a 21-item questionnaire and will be used to measure parent emotional well-being at baseline, before randomisation and at all time points thereafter.

#### Bayley Scales of Infant and Toddler Development Third Edition (BSID-III)

The BSID-III [47] is a standardised and norm referenced assessment, which measures the cognitive, motor, language and social-emotional development of infants and toddlers aged 0–3. It consists of a number of developmental play tasks that can be completed at the child’s home and videoed for scoring by blind raters. Alternatively infants enrolled in follow up programmes from recruitment sites may be assessed by staff blinded to group allocation at their 1- year clinic appointment. Infants will be assessed on the BSID-III at 12 months and 2 years.

### Statistical methods

Analysis will be conducted on an intention-to-treat basis using SPSS and reported according to the CONSORT statement. Descriptive statistics (frequencies, means and 95% CIs) will be used to describe the sample at baseline and data from each outcome measure used will be summarised for both treatment groups. Between-group differences following intervention will be analysed using multiple regression to determine whether group allocation predicts outcome. MRI classification, SES and comorbidities including vision impairment and epilepsy will be considered as covariates in the analysis.

## Discussion

This paper outlines the design and background for a single blind RCT comparing a novel intervention “GAME” with standard care to improve the motor outcomes of infants at high risk of CP.

## Appendix

### Study Sites

1. Cerebral Palsy Alliance, NSW Australia
2. Sydney Childrens Hospital Network, NSW Australia
3. Royal Prince Alfred Hospital, NSW Australia
4. Westmead Hospital, NSW Australia
5. Royal North Shore Hospital, NSW Australia
6. Liverpool Hospital, NSW Australia
7. Royal Women’s Hospital, NSW Australia

## Abbreviations

CP: Cerebral palsy
GMs: General movements
EI: Early intervention
EE: Environmental enrichment
GAME: Goals-Activity-Motor-Enrichment
HP: Home program
PDMS-II: Peabody Developmental Motor Scale -2^nd^ Edition
GMFM: Gross Motor Function Measure
COPM: Canadian Occupational Performance Measure
BSID-III: Bayley Scales of Infant and Toddler Development- 3^rd^ Edition
AHEMD-IS: Affordances in the Home Environment for Motor Development – Infant version
DASS-21: Depression, Anxiety and Stress Scale – 21 version
